# Aspergillosis in Chronic Granulomatous Disease

**DOI:** 10.3390/jof2020015

**Published:** 2016-05-26

**Authors:** Jill King, Stefanie S. V. Henriet, Adilia Warris

**Affiliations:** 1 Aberdeen Fungal Group, MRC Centre for Medical Mycology, Institute of Medical Sciences, University of Aberdeen, Aberdeen AB25 2ZD, UK; j.king@abdn.ac.uk; 2 Radboud University Medical Center, Amalia Children’s Hospital, Nijmegen 6500 HB, The Netherlands; Stefanie.Henriet@radboudumc.nl

**Keywords:** aspergillosis, chronic granulomatous disease, *Aspergillus fumigatus*, *Aspergillus nidulans*, NADPH-oxidase, respiratory burst

## Abstract

Patients with chronic granulomatous disease (CGD) have the highest life-time incidence of invasive aspergillosis and despite the availability of antifungal prophylaxis, infections by *Aspergillus* species remain the single most common infectious cause of death in CGD. Recent developments in curative treatment options, such as haematopoietic stem cell transplantation, will change the prevalence of infectious complications including invasive aspergillosis in CGD patients. However, invasive aspergillosis in a previously healthy host is often the first presenting feature of this primary immunodeficiency. Recognizing the characteristic clinical presentation and understanding how to diagnose and treat invasive aspergillosis in CGD is of utmost relevance to improve clinical outcomes. Significant differences exist in fungal epidemiology, clinical signs and symptoms, and the usefulness of non-culture based diagnostic tools between the CGD host and neutropenic patients, reflecting underlying differences in the pathogenesis of invasive aspergillosis shaped by the nicotinamide adenine dinucleotide phosphate (NADPH)-oxidase deficiency.

## 1. Introduction

### 1.1. Epidemiology of Invasive Aspergillosis in CGD

Patients with chronic granulomatous disease (CGD) have the highest life-time incidence of invasive aspergillosis (IA) and despite the availability of antifungal prophylaxis and targeted antifungal therapy, *Aspergillus* species remain the most common cause of infectious complications [1,2,3]. CGD is a genetically heterogeneous disease caused by a defect in any of the five structural components of the nicotinamide adenine dinucleotide phosphate (NAPDH) oxidase complex including the granule or plasma membrane-bound glycoproteins gp91^phox (phagocyte oxidase)^ and p22^phox^, and the cytoplasmatic components p47^phox^, p67^phox^ and p40^phox^ [4]. Up to now, five genes are responsible for all known cases of CGD: The X-linked inherited *CYBB*, and the autosomal recessive *CYBA*, *NCF1*, *NCF2* and *NCF4*, respectively [5]. Incidence ranges from 1/250,000 in the US to 1/120,000 in the UK and Ireland with the prevalence of recessive forms varying depending on consanguinity rates in particular countries [6,7,8,9,10]. The defect in the NADPH-oxidase leads to a failure to generate a proper phagocyte respiratory burst and subsequent superoxide production. The NADPH-oxidase complex plays an important role in signaling pathways leading to inflammation and immunity [11]. The amount of residual superoxide production correlates with the underlying genotype and is a strong predictor of overall survival [12]. 

The epidemiology of IA in CGD patients differs from other immunocompromised patient groups with regards to the incidence of IA, the causative *Aspergillus* species, the localization of *Aspergillus* infections and the impact of IA on overall survival in this patient group [13]. Reported incidence of IA ranges from 26% to 45% with lungs, bones and brain being the most affected organs [3,13]. By reviewing the published data of CGD patient registries, it can be concluded that *Aspergillus* species are the most commonly isolated organisms in case of pulmonary infection, osteomyelitis and focal brain infection [3,13]. The vast majority of IA in CGD is caused by either *Aspergillus fumigatus* or *Aspergillus nidulans*. The latter species demonstrates a unique interaction with the CGD host and is hardly reported to cause IA in other immunocompromised patients [2,13,14]. The exact fungal epidemiology is not well-known due to a lack of isolation of the causative fungal pathogen and the identification to species level. It is estimated that 55% of IA in CGD is caused by *A. fumigatus* and about 35% by *A. nidulans* (Table 1) with a higher proportion of *A. nidulans* infections observed in osteomyelitis and those patients receiving itraconazole prophylaxis [2,13,15]. *Aspergillus*-specific mortality seems to show a decreasing trend over time from around 30% [13] to below 20% [2,3] although pulmonary IA is still the main cause of infectious death in CGD patients. 

A clear association between the particular defect in the NADPH-oxidase and the extent to which the superoxide production is affected, and the occurrence and severity of IA can be observed. X-linked (gp91^phox^) CGD patients have both a higher incidence (Table 1) and worse outcome of IA compared to those with autosomal recessive CGD [3,13]. Median age of first *Aspergillus* infection is lower for patients with X-linked CGD compared to those with the autosomal recessive form (Table 1). Interestingly, a higher prevalence of IA caused by *A. nidulans* is observed in X-linked CGD (Table 1). However, Marciano *et al.* showed that the residual activity of the NADPH-oxidase complex, and not the genetic defect *per se*, determines the risk of IA and severe infections in CGD [3]. When categorizing patients based on residual superoxide production, incidence of IA was 55% in the lower superoxide quartiles compared to 41% in the higher superoxide production quartiles [3].

### 1.2. Prophylactic Measures

The high life-time incidence of IA in CGD and its direct association with a shortened life-span in CGD patients, indicates a clear role for preventive measures. Prevention of IA plays a central role in the clinical management of CGD patients and consists of reducing environmental exposure to moulds and the prophylactic use of antifungals. Exposure to mulch, hay, wood chips and rotting plants (compost heaps), visiting caves, stables, sheds and areas of construction and/or renovation, and activities such as gardening should be avoided [16].

The use of itraconazole prophylaxis has been shown to significantly reduce invasive fungal functions in CGD patients [17,18]. Posaconazole, a newer mould-active azole with a broader spectrum of activity and increased tolerability, seems to be a favourable alternative [19]. Nevertheless, prescription of antifungal prophylaxis varies widely between countries with as low as 48% in Japan to 93% in the UK [8,20,21,22]. Additionally, in the most recent published large CGD case series, antifungal prophylaxis was used by only 70% of patients [3]. The reason for this is not clear, but consideration of possible toxicity and adverse events related to antifungal prophylaxis may play a role. Prolonged antifungal prophylaxis may lead to changes in the fungal epidemiology and antifungal resistance [23,24]. However, this has not been shown so far on a large scale in this particular patient group.

The use of prophylactic recombinant human interferon-γ (IFN-γ) has shown to decrease the risk of severe infections (including fungal infections) in CGD by 70% [25]. However, controversy remains about its use in routine prophylaxis [26,27]. In the most recent case series, IFN-γ prophylaxis is reported in about 27% to 40% of CGD patients with slightly higher use in the US compared to Europe [3,13,20]. Of important to note, most studies with IFN-γ in CGD were performed before modern antifungal prophylaxis was standard of care, and it remains to be seen if IFN-γ has an additive role in the prevention of IA next to prophylaxis with antifungals. 

## 2. Diagnosis of Invasive Aspergillosis

### 2.1. Clinical Presentation

The clinical presentation of IA in CGD can be highly variable, is often indolent with minimal symptoms, and may only be detected during routine screening, highlighting the importance of maintaining a high index of clinical suspicion. Invasive aspergillosis may also represent the primary presenting feature of CGD and the identification of IA in a patient with no known risk factors for fungal infection should prompt further investigation.

Henriet *et al.* recently reviewed all published cases and case series’ of invasive fungal infection in CGD between 1970 and 2010 [13]. This has allowed a better understanding of the clinical presentation and diagnostic features of IA specific to CGD. Presentation varies by site of infection and by infecting organism. 

Fever is common in *A. fumigatus* infection, reported in 61%, but it is not a universal presenting feature and the absence of fever does not preclude infection [13]. A recent review of a French cohort of CGD patients with invasive fungal infection found 37% reported neither fever nor respiratory symptoms at the time of diagnosis [28]. This is similar to the 1/3^rd^ of patients with IA presenting to the NIH who were asymptomatic at the time of diagnosis [29]. In a 25-year retrospective survey looking at invasive fungal infection in children with CGD registered to the French National Database for Primary Immunodeficiency, failure to thrive was the most common presenting feature, present in 71% [2].

It is not surprising given the ubiquitous environmental presence of *Aspergillus* conidia, and our capacity to inhale thousands of spores each day [30], that the primary site of infection in the majority of patients is pulmonary. Pulmonary aspergillosis in CGD is typified by a chronic, progressive pneumonia. Respiratory symptoms may include chest discomfort, cough (usually non-productive), and progressive dyspnea [13]. In contrast to neutropenic patients, haemoptysis is rarely reported in CGD, reflecting the differing pathogenesis of IA in the neutropenic (angio-invasive) *vs.* the non-neutropenic host [13,29,31]. 

The clinical presentation of *A. nidulans* infection is often more subtle and non-specific than *A. fumigatus* infection. Symptoms, when present, include low-grade fever, localized pain or swelling, non-productive cough, and general malaise [13]. In contrast to this benign presentation, 74% of primary pulmonary infections caused by *A. nidulans* are complicated by extensive tissue destruction and direct spread to adjacent structures [13]. It is unclear whether this represents delayed presentation due to lack of symptoms or a more aggressive disease pathogenesis.

In addition to the typical chronic, progressive pneumonia seen in the majority of patients, pulmonary aspergillosis may present with an acute, life-threatening pneumonitis known as ‘mulch pneumonitis’. This typically follows exposure to high levels of aerosolized conidia during gardening activities such as lawn care or applying leaf mulch [13,31]. Symptoms are of an acute pneumonia with severe hypoxia and, despite aggressive management with antifungals, high dose steroids, mechanical ventilation and ECMO (extracorporeal membrane oxygenation), mortality is high (73%). This unusual presentation appears unique to CGD and in 36% of reported cases it was the primary presenting feature leading to a diagnosis of CGD [13].

The second most common site of *Aspergillus* infection in CGD is the skeleton. CGD is the most common primary immunodeficiency associated with *Aspergillus* osteomyelitis and *Aspergillus spp.* are the most common organisms causing osteomyelitis in CGD [19,32]. Dissemination of pulmonary IA to the skeleton has been described in 35% of CGD patients, and in 65% of CGD patients with *A. nidulans* infection [13].

In contrast to an earlier study where *A. nidulans* osteomyelitis was found more frequently than osteomyelitis caused by *A. fumigatus* [33], a recent study by Dotis and Roilides found an almost equal frequency of infection with each organism [15]. However, *A. nidulans* has a greater propensity to cause osteomyelitis, with 74% of infections complicated by osteomyelitis compared to 41% of *A. fumigatus* infections [13]. In the study by Dotis and Roilides, *A. nidulans* osteomyelitis cases occurred exclusively in male patients and, where the genotype was known, all patients carried the gp91^phox^ mutation [15].

Bony lesions are typically multifocal and thoracic vertebrae and ribs are the most commonly affected sites [15,34]. This reflects the pathogenesis of infection through direct spread from an adjacent pulmonary focus rather than via haematogeonous dissemination. Haematogenous dissemination is however possible, particularly with *A. fumigatus* infection, and isolated osteomyelitis has also been reported in CGD [13,15,32]. Clinical features have not been well described but the most common manifestations appear to be localized pain and tenderness, often without fever. 

Localized brain abscesses are considered rare but the study by van den Berg *et al.*, looking at 429 European CGD patients, found that 7% had at least one episode of brain abscess, 38% of which were due to *Aspergillus* spp. [20]. This is similar to the frequency reported by Bortoletto *et al.* in their smaller, single centre retrospective cohort [35] although higher than the 4% recorded in the US CGD Registry data [7]. It is worth noting that isolated brain abscess has been reported as the primary presenting feature of CGD in several published case reports including a 16-year-old boy investigated for possible intracranial tumour [36,37]. In all studies *Aspergillus* spp. were the most common causative agents of brain abscess in CGD [7,20,35]. Clinical features vary from mild fever and headaches to seizures and localizing signs mimicking intracranial tumour [13]. Vertebral invasion is associated with signs of spinal cord invasion in 45% [13].

Other less common sites of infection include skin, lymph nodes, liver and spleen [13,20]. Skin infections are generally preceded by superficial abrasion or minor trauma. The clinical manifestations are diverse, from erythematous plaques and papules to pustules and purulent ulcers mainly localized to the extremities. Secondary cutaneous infection due to haematogenous spread is an aggressive clinical feature associated with high mortality [13].

Hepatic and splenic abscesses due to *Aspergillus* spp. are typically seen during disseminated infection rather than occurring in isolation [13]. Case reports of more unusual presentations of IA in CGD include endocarditis secondary to *A. nidulans* infection in a three-year-old girl with CGD and an atrial septal defect [38], and a report of pericarditis in a 40-year-old woman with CGD presenting with increasing dyspnea [39].

### 2.2. Laboratory Findings

There is limited information available regarding typical laboratory findings in IA in CGD. Leukocytosis is often absent, with over half (13/23) of patients in one series having a white blood cell count ≤ 10,000 μL [29,31]. Erythrocyte sedimentation rate (ESR) is frequently elevated in CGD patients due to other inflammatory pathology and is not particularly helpful in distinguishing infection [31]. Even in the presence of IA, Segal *et al.* found almost half of CGD cases (9/20) had an ESR ≤ 40 mm/h further limiting its usefulness as a diagnostic marker [26]. CRP may be more helpful as it is usually not elevated in CGD patients in the absence of infection [28,31]. However, given the minimal systemic inflammatory response despite significant infection it remains to be seen if CRP is a useful marker of IA in CGD.

### 2.3. Imaging

Imaging helps to localize and assess the extent of infection. Chest X-ray (CXR) changes in the CGD host are typically non-specific, with segmental or multi-lobar consolidation, peri-hilar infiltrates, small nodules, or pleural effusions the most common findings [13,40]. While nodular changes are more suggestive of invasive fungal infection, there are no specific CXR findings that help to distinguish IA from other causes of pneumonia in CGD [13].

Computed tomography (CT) has become the mainstay for investigating possible invasive fungal infection in CGD due to its utility in delineating the extent of soft tissue damage and bony involvement. However, there have been no large systematic reviews of radiological findings in IA in CGD. The typical findings of cavitation, pulmonary infarction and the air crescent or halo signs seen in neutropenic patients are not found in CGD. Local extension from lung parenchyma to adjacent structures and osteomyelitis of the thoracic cage are findings that are particularly associated with IA in CGD [13,40,41].

Osteomyelitis in CGD is typified by extensive destruction and osteolysis with extension into adjacent soft tissue. Infection is often multi-focal and sclerosis, which is considered a late change, may be present at the time of diagnosis due to delayed presentation [13,40,41]. 

Isolated *Aspergillus* brain abscesses are typically seen as either single or multiple ring-enhancing lesions primarily located at the grey–white matter junction on MRI (magnetic resonance imaging) or contrast-enhanced CT [40,41]. 

### 2.4. Biopsy and Culture

In contrast to neutropenic patients, CGD patients with aspergillosis are usually in a reasonable condition and clinically stable, therefore every effort should be made to identify the infecting organism before initiating treatment. Histopathology and culture are complimentary and allow accurate identification of the infecting organism and antifungal susceptibility testing. Fungal elements seen in pathology specimens may assist in the identification of the infecting organism however different fungal species can appear morphologically very similar and identification should not rely on histopathology alone [41]. Both histopathological evidence of invasive tissue growth and positive culture are required for proven infection in the EORTC/MSG (European Organisation for Research and Treatment of Cancer/Mycoses Study Group) definitions of invasive fungal infection [42]. 

Culture confirmation is important in differentiating *Aspergillus* spp. from other filamentous fungi, assisting with identification to the species level, and allowing antifungal susceptibility testing [43,44]. The importance of identification to species level is highlighted by the inherent reduced susceptibility of *A. nidulans* to amphotericin B [45,46,47] and the relative azole resistance of recently identified organisms such as *A. tanneri* [48] and *A. udagawae* [49].

Broncho-alveolar lavage (BAL), fine needle aspiration (FNA), trans-thoracic percutaneous biopsy or video-assisted thorascopic (VAT) biopsy are among the techniques used to diagnose pulmonary IA [44]. The reported yield in CGD patients with IA varies from 12.5% to 52% and likely depends on the localization of pulmonary abnormalities and BAL technique [1,3,28]. Lung biopsy increased the pathogen detection rate from 30% to 50% in CGD patients and is preferred to enable an aetiological diagnosis.

Determining the sensitivity of FNA and percutaneous biopsy in CGD is difficult due to the small number of reported cases. In their review of all reported cases of invasive fungal infection in CGD, Henriet *et al.* found fine needle lung biopsies in CGD patients with invasive fungal infection were all positive, either on microscopy or culture [13]. However, in the study by Blumental *et al.*, only 21% of pulmonary FNAs were positive, with the majority of cases confirmed by surgical biopsy [2]. The localization of infection, accessibility to percutaneous sampling, and use of imaging to assist with obtaining samples all likely impact on the diagnostic yield.

Galluzzo *et al.* performed an interesting study in which biopsies from osteomyelitis in CGD patients were compared with those from patients without CGD [34]. In addition to their ability to identify the causative pathogen in all biopsies taken (42% *Aspergillus* species), clear histopathological differences between CGD and non-CGD patients were observed. In CGD patients, chronic inflammation plus granulomata, multinucleated giant cells, histiocytes and/or necrosis were significantly overrepresented compared to non-CGD patients. The presence of granulation tissue, remodeled bone or lymphocytes was significantly underrepresented in CGD patients [34]. 

Extracting the results from the large cohort of European CGD patients described by van den Bergh *et al*., it can be concluded that a causative diagnosis is not being made in the majority of CGD patients presenting with a localized infection [20]. It is not possible to determine the reasons for this, either no cultures (e.g., biopsy, BAL, blood) were taken or cultures remained negative. An exception was CGD patients with liver abscesses who underwent a biopsy where culture remained negative in 70%. 

### 2.5. Non-Culture Based Diagnostics

Non-culture based diagnostic test have been developed to assist in the screening of high-risk neutropenic patients and facilitate early diagnosis of IA followed by prompt pre-emptive therapy to improve outcome. 

Galactomannan, a polysaccharide present in the *Aspergillus* cell wall during active growth, can be detected in serum, BAL, cerebrospinal fluid (CSF) and other body fluids [43]. A recent Cochrane review demonstrated a sensitivity and specificity of 82% and 81% respectively in neutropenic patients [50]. However, circulating galactomannan is detected in only a quarter of non-neutropenic patients and is most likely influenced by the absence of angio-invasive growth in this patient group. In published case series’ where serum galactomannan is reported, the majority of CGD patients remain negative despite confirmed *Aspergillus* infection [1,2,13,28]. 

Although the underlying pathogenesis of IA in CGD will explain the lack of galactomannan in serum, the use of antifungal prophylaxis and removal of galactomannan by neutrophils and other immune processes may also contribute [51,52]. Galactomannan testing in BAL specimens is widely used in high-risk neutropenic patients and a systematic review and meta-analysis found BAL galactomannan had greater sensitivity than serum galactomannan in diagnosing IA [53]. A study performed among patients, majority (78%) non-neutropenic, admitted to the ICU, showed the usefulness of BAL galactomannan in the diagnosis of IA with a high sensitivity (88%) and specificity (87%) [54]. In the same study, sensitivity of serum galactomannan was only 42%. Although there have been no specific reports on the use of BAL galactomannan in CGD patients, galactomannan testing in BAL may assist in diagnosis of IA in this patient group. In line with this, FNAs from localized abscesses in the CGD host may be submitted for galactomannan analysis as it has been shown positive results can be obtained [55]. Use of the recently developed *Aspergillus* lateral-flow device on BAL samples shows promising results for the diagnosis of IA in non-neutropenic ICU patients with comparable sensitivity and specificity to BAL galactomannan [56]. This point-of-care test uses a monoclonal antibody to detect an extracellular mannoprotein antigen secreted exclusively by *Aspergillus* species.

PCR as a biomarker to detect *Aspergillus* in serum or plasma is of limited use in CGD patients for the same reasons discussed in galactomannan detection. In CGD cases with reported PCR results, serum and CSF samples were negative, however biopsy and sputum specimens have shown positivity [13,57]. In addition, PCR can be designed to identify to a species level as well as detecting mutations conferring resistance to specific antifungals (e.g., azole resistance in *A. fumigatus*) [58]. 

## 3. Treatment

### 3.1. Antifungals

Since the pivotal trial by Herbrecht *et al.* demonstrated the superiority of voriconazole over amphotericin B deoxycholate [59], voriconazole has been the recommended first line antifungal agent for IA [44]. This triazole antifungal agent is active against both *A. fumigatus* and *A. nidulans*, the most frequent fungal pathogens observed in CGD, and has good central nervous system (CNS) penetration making it the first line of treatment as well in CNS aspergillosis. However voriconazole is not without limitations; in keeping with other azoles the pharmacokinetics are unpredictable and non-linear, necessitating therapeutic drug monitoring (TDM); short and long-term side-effects including visual disturbances; significant drug interactions; solar hypersensitivity and the risk of skin malignancy [44,60,61,62].

There has been significant interest in using newer azole antifungal agents in IA, either as rescue therapy, or as primary therapeutic agents. Posaconazole has been shown to be safe and effective in CGD patients with IA refractory to conventional antifungal therapy and has good activity against both *A. fumigatus* and *A. nidulans* [63,64,65]. Optimum paediatric dosages for treatment with posaconazole have not been well established, in particular in young infants, and published case series have focused on plasma levels during posaconazole prophylaxis of which only one was performed in paediatric CGD patients [19,66,67,68]. The relationship between dosages and plasma levels obtained can be used as a guide to direct therapeutic use of posaconazole [19]. A lack of a parenteral preparation and the time taken to reach steady state (up to one week) may limit the use of posaconazole as primary therapy.

Another promising azole antifungal, isavuconazole, has recently been shown to be non-inferior to voriconazole in the primary treatment of IA or other invasive mould infections in adult allogeneic haematopoietic stem-cell transplantation (HSCT) patients and adult patients with haematological malignancies (SECURE trial) [69]. Fewer drug-related adverse events were seen in the isavuconazole group suggesting it may be a suitable alternative for patients unable to tolerate voriconazole. The availability of both an oral and intravenous formulation gives it clear advantages compared to posaconazole in the (initial) treatment of IA. At present, there are no data about dosing and safety in children, and no recommendations can be made how to use this new azole antifungal in children with CGD.

An impending and significant challenge in the treatment of IA is the emerging acquired azole resistance observed in *Aspergillus* isolates in addition to *Aspergillus* species being intrinsically resistant or less susceptible to azoles [47,70]. Multi- or pan-azole resistance appears more common than resistance to a single agent and liposomal amphotericin B (L-AmB) is recommended as primary therapy when azole resistant *Aspergillus* isolates are identified [71]. Alternative treatment options include combination therapy with voriconazole and L-AmB or class switching to an echinocandin [72].

In addition to antifungal treatment, combination with extensive and early surgical debridement is reported to be used in most CGD patients with IA [2,13,15]. In a multi-centre retrospective analysis of paediatric IA, with a variety of underlying conditions, no specific antifungal regimen was found to be superior to the other. The only intervention with a positive effect on mortality was surgery [72]. Similarly, in a review of *Aspergillus* osteomyelitis by Gamaletsou *et al.* where CGD patients represented 15% of all cases and 73% of paediatric cases, a significantly reduced rate of relapse in those treated with a combination of medical and surgical therapy *vs.* medical therapy alone (8% *vs.* 30% respectively, *p* = 0.006) was observed [32].

Another aspect of treatment with limited evidence to support practice is the management of IA in CGD patients taking regular antifungal prophylaxis, which all CGD patients should be receiving. It is unclear if breakthrough infections represent inadequate plasma azole concentrations due to their highly variable pharmacokinetics, non-adherence, the development of azole resistance, or infections with fungal pathogens intrinsically resistant to azoles. More work is needed to better understand breakthrough infections in CGD. Ensuring appropriate clinical sampling is required to identify the causative fungus at the species level and enable antifungal susceptibility testing to direct targeted antifungal therapy.

### 3.2. Immunomodulatory Treatments

Different adjunctive immunomodulatory treatment options have been suggested in patients suffering from CGD and severe infections. The use of granulocyte transfusions in CGD is supported by the observation that *in vitro* a small number of normal phagocytes may complement the oxidative defect and restore the killing ability towards *A. fumigatus* hyphae [73]. In a comprehensive overview of all published invasive fungal infections in CGD patients from 1970–2010, 22% of the 116 probable and proven invasive fungal infections were treated with granulocyte transfusion [13]. However, the efficacy of granulocyte transfusions is poorly documented, and especially uncertain and unpredictable in patients who become allo-immunized [74,75]. Allo-immunization to both human leucocyte antigens (HLA) and neutrophil-specific epitopes occurs in up to 70% of CGD patients who have received granulocyte transfusions, and these antibodies have been shown to destroy transfused granulocytes [76,77]. The dihydrorhodamine 123 (DHR) assay provides a valuable means to monitor the viability of the donor granulocytes which is strongly correlated with the absence of allo-immunization. If survival of donor granulocytes is low, e.g., proportion of granulocytes with intact oxidase activity is low, transfusions should be avoided [75]. It should be kept in mind that allo-immunization prior to HSCT might prove detrimental to neutrophil recovery.

Interferon-γ (IFN-γ) is a macrophage activating cytokine produced by T cells and natural killer cells, and has a key role in the innate and adaptive host response against fungi [78]. IFN-γ is a FDA-approved agent to reduce the frequency and severity of infections in CGD patients. A subgroup of variant X-linked CGD patients (*i.e*., with very low, but detectable, baseline superoxide-generating activity), who have splice site mutations, has been shown to be responsive to IFN-γ [79,80]. Treatment resulted in improved splicing efficiency and an increase in cytochrome b expression, allowing near normal levels of superoxide production and bactericidal activity of neutrophils and monocytes [81,82,83]. However, subsequent reports have not confirmed the enhancement of superoxide production as the principal mechanism of IFN-γ activity in CGD patients [84,85]. Additionally, there are no prospective studies to show any benefit of IFN-γ treatment during acute infection.

## 4. Future Perspectives

### 4.1. Immunomodulatory Therapy

The fast improving insights in the molecular interaction of *Aspergillus* species and the CGD host has the potential to result in the design of new management strategies. Hyperinflammation is the Achilles heel of the CGD host, and IA in CGD seems to be not only the result of a defective clearing of the fungal pathogen, but also of the enormous host damage due to exaggerated host inflammatory response. These findings have major implications in the direction of new treatment strategies. Strategies need to target the dysregulated inflammation which is at least partly responsible for the induced host damage and detrimental outcome of invasive *Aspergillus* infections in the CGD host. Consequently, medical treatments aimed at killing the fungus (e.g., antifungal drugs) are expected to incompletely target the disease process.

Recently, we showed that the old malarial drug chloroquine has antifungal properties and dampens the inflammatory response of CGD cells *in vitro* [86]. Chloroquine targets both limbs of fungal pathogenesis and may be of great value in the treatment of IA in patients with CGD. Evaluation of the antifungal and anti-inflammatory potential of the less toxic chloroquine compounds (e.g., hydroxychloroquine) is now needed. Furthermore, *in vivo* studies using experimental models of IA are necessary to validate the *in vitro* observations before these findings can be translated into clinical practice. Hydroxychloroquine has already shown promise in the treatment of granulomatous complications in CGD, probably by reduction of the inflammatory mediators implicated in granuloma formation [87]. Moreover, in contrast to corticosteroids commonly used in the treatment of granulomata, hydroxychloroquine is a drug that does not increase the risk of infectious adverse events. 

Hypothesizing that hyperinflammation is largely responsible for the disease severity of IA in CGD, Anakinra^®^, an IL-1-receptor antagonist (IL-1Ra), may be a potential additive treatment option. It is widely used in the management of rheumatoid arthritis and has shown efficacy in the treatment of inflammatory complications in CGD patients [88,89,90,91]. Furthermore, Anakinra^®^ can increase phagosome maturation in CGD phagocytes, resulting in inhibition of fungal growth, dampening the IL-1β release [91]. In the same paper, the authors observed that Anakinra^®^ may improve the outcome of experimental IA caused by *A. fumigatus* in CGD mice with the autosomal recessive variant of CGD (p47^phox−/−^mice).

The role of the IL-1α/G-CSF axis in inflammation in CGD has recently been identified and highlighted as a potential target for immunomodulatory treatment. NADPH-deficiency enhances the early local release of IL-1α in response to damaged cells, promoting an excessive G-CSF regulated neutrophilic response and prolonged inflammation. Higher levels of systemic G-CSF increased peripheral neutrophilia, which amplified neutrophilic peritoneal inflammation in X-CGD mice. Dampening early neutrophil recruitment by neutralization of IL-1α, G-CSF, or neutrophil depletion itself promoted resolution of otherwise prolonged inflammation in X-CGD [92]. Further research into the role of IL-1α and IA in CGD patient is needed to be done before any further conclusions can be made on the therapeutic role of intervening with the IL-1α/G-CSF axis.

The galactosaminogalactan (GAG) is a cell wall component of *A. fumigatus* that has potent anti-inflammatory effects in mice and humans due to its capability to induce IL-1Ra. The capacity of GAG to induce IL-1Ra seems an interesting lead to modulate diseases characterized by hyperinflammation, as GAG was shown to reduce the severity of allergic aspergillosis colitis in experimental models of disease [93]. Induction of IL-1Ra by GAG might prove beneficial as an immunomodulatory agent in the treatment of IA but further studies are awaited.

### 4.2. Haematopoietic Stem-Cell Transplantation and Gene Therapy

To date, haematopoietic stem-cell transplantation (HSCT) is the only curative option for patients with CGD [94], and has also been used to treat refractory invasive fungal infections in CGD with variable outcome [95]. The complexities of the clinical phenotype of CGD, on one hand, and the perceived and real risks associated with HSCT, on the other, have prevented many patients from getting this curative treatment. Recent advances in HSCT techniques have shown this option is much safer than previously thought and that a successful procedure is not only curative but restores the quality of life to normal [96]. Gene therapy, looking promising in several single gene primary immunodeficiencies, such as severe combined immunodeficiencies (SCID), is currently not an available treatment option due to the need for improved and safer vectors, but is likely to have a more significant role in the future [97]. Although functional correction of only a minor fraction of the neutrophils (approximately 10%) provides complete clinical protection, as learned from X-linked CGD carriers, the absence of growth or survival advantage of the transduced stem cells in the marrow or in the tissue is a point of significant concern [98].

## 5. Summarizing Remarks

Life expectancy for patients with CGD has increased, largely due to universal antibacterial and antifungal prophylaxis and increased awareness of infectious complications. Despite the development and availability of a number of oral mould-active azole antifungals in the last decades, a substantial number of CGD patients appear not to benefit from it. Initial clinical signs and symptoms of IA are often indolent and a high clinical suspicion in CGD patients is important to enable early treatment, improve outcome and prevent death. Recent studies indicate that the pathophysiology of IA in the CGD host is mainly determined by the hyperinflammation evoked by *Aspergillus*. Immunomodulatory treatment should be targeted at decreasing the hyperinflammation thereby preventing organ damage and improving outcome both in terms of morbidity and mortality. Importantly, the immunomodulatory treatment should not negatively interfere with these antifungal immune responses which are not affected by the absence of a functional NADPH-oxidase complex. As long as cure is not feasible for the majority of patients with CGD, optimizing the management of IA, the main cause of premature death, needs to be aimed for.

## Figures and Tables

**Table 1:** Clinical epidemiology of invasive aspergillosis caused by *A. fumigatus* and *A. nidulans* in chronic granulomatous disease patients.

	Blumenthal 2011 [2]	Dotis 2011 [15] *	Beaute 2011 [1]	Henriet 2013 [13]	Marciano 2015 [3]
**CGD patients with IA**	19	43	32	60	125
**IA in XL-CGD**	11 (58%)	14 (78%)	NA	38 (63%)	90 (72%)
**Median age of first IA in XL-CGD**	6 years (0.5–17 years)	NA	NA	10 years (0–32 years)	12 years (0.1–38 years)
**IA in AR-CGD**	6 (32%)	4 (22%)	NA	8 (13%)	35 (28%)
**Median age of first IA in AR-CGD**	7.5 years (0.5–14 years)	NA	NA	16 years (4.5–64 years)	17 years (4–62 years)
**IA caused by *A. fumigatus*/*A. nidulans***	10(59%)/7(41%)	21(53%)/19(47%)	14(56%)/11(44%)	37(62%)/23(38%)	NA
**In XL-CGD**	2(20%)/8(80%)	9(43%)/12(57%)	NA	25(60%)/17(40%)	NA
**In AR-CGD**	4(67%)/2(33%)	3(75%)/1(25%)	NA	8(80%)/2(20%)	NA
**Median age of first *A. fumigatus* infection**	2 years (0.5–8 years)	9 years (3–20 years)	NA	10 years (0–64 years)	NA
**Median age of first *A. nidulans* infection**	9 years (2–17 years)	8 years (1.5–21 years)	NA	8 years (3–21 years)	NA

CGD, chronic granulomatous disease; IA, invasive aspergillosis; XL, X-linked; AR, autosomal recessive; NA, data not available; * *Aspergillus* osteomyelitis only.
